# Heat stress during male meiosis impairs cytoskeletal organization, spindle assembly and tapetum degeneration in wheat

**DOI:** 10.3389/fpls.2023.1314021

**Published:** 2024-01-08

**Authors:** Attila Fábián, Barbara Krárné Péntek, Vilmos Soós, László Sági

**Affiliations:** ^1^ Centre for Agricultural Research, Hungarian Research Network, Martonvásár, Hungary; ^2^ Department of Applied Biotechnology and Food Science, Budapest University of Technology and Economics, Budapest, Hungary; ^3^ Institute of Genetics and Biotechnology, Hungarian University of Agriculture and Life Sciences, Gödöllő, Hungary; ^4^ Agribiotechnology and Precision Breeding for Food Security National Laboratory, Plant Biotechnology Section, Centre for Agricultural Research, Hungarian Research Network, Martonvásár, Hungary

**Keywords:** cytoskeleton, gene expression, heat stress, meiosis, RNA-seq, spindle apparatus, tapetum, *Triticum aestivum*

## Abstract

The significance of heat stress in agriculture is ever-increasing with the progress of global climate changes. Due to a negative effect on the yield of staple crops, including wheat, the impairment of plant reproductive development triggered by high ambient temperature became a restraint in food production. Although the heat sensitivity of male meiosis and the following gamete development in wheat has long been recognized, a detailed structural characterization combined with a comprehensive gene expression analysis has not been done about this phenomenon. We demonstrate here that heat stress severely alters the cytoskeletal configuration, triggers the failure of meiotic division in wheat. Moreover, it changes the expression of genes related to gamete development in male meiocytes and the tapetum layer in a genotype-dependent manner. ‘Ellvis’, a heat-tolerant winter wheat cultivar, showed high spikelet fertility rate and only scarce structural aberrations upon exposure to high temperature. In addition, heat shock genes and genes involved in scavenging reactive oxygen species were significantly upregulated in ‘Ellvis’, and the expression of meiosis-specific and major developmental genes showed high stability in this cultivar. In the heat-sensitive ‘Mv 17-09’, however, genes participating in cytoskeletal fiber nucleation, the spindle assembly checkpoint genes, and tapetum-specific developmental regulators were downregulated. These alterations may be related to the decreased cytoskeleton content, frequent micronuclei formation, and the erroneous persistence of the tapetum layer observed in the sensitive genotype. Our results suggest that understanding the heat-sensitive regulation of these gene functions would be an essential contribution to the development of new, heat-tolerant cultivars.

## Introduction

The ongoing global warming exerts an increasingly negative impact on agriculture, decreasing the realized yield of crop plants, including wheat, worldwide (Pachauri et al., 2014; Lesk et al., 2016). The reproductive development of plants is especially vulnerable to extreme temperature stress (Farooq et al., 2011; Ullah et al., 2022), also supported by current models that predict a serious decrease in the global yield of wheat due to waves of high ambient temperatures during the vegetation period (Sun et al., 2019). Disturbances during early reproductive development can frequently lead to a decreased seed number, with evident yield penalties (Dolferus et al., 2011). Since winter wheat (*Triticum aestivum* L.) is one of the most important staple crops, characterization of its heat stress response and the improvement of tolerance will be a priority task for plant biologists and breeders for the next decades (Stratonovitch and Semenov, 2015). Literature data indicate that the male gametophyte exhibits higher sensitivity to high temperatures when compared to the female counterpart (Hedhly, 2011; Giorno et al., 2013). However, our previous results showed that pre-anthesis heat stress combined with drought severely impairs the function of wheat stigma papilla cells, decreasing spikelet fertility and, subsequently, the grain yield (Fábián et al., 2019).

Plants, being sessile organisms, are obliged to adapt to various environmental factors, such as heat stress. Negative impacts of high temperature are alleviated by the heat stress response (HSR) (Chaturvedi et al., 2021). HSR includes the activation of heat shock transcription factors (HSF), which induce the expression of heat shock proteins (HSP) (Haider et al., 2022). These proteins contribute to the alleviation of damage induced by high temperature stress and various other abiotic and biotic stress factors (ul Haq et al., 2019). Antioxidant molecules and enzymes are also substantial part of the HSR, lowering the amount of reactive oxygen species triggered by the high temperature (Medina et al., 2021). In the case of wheat, several studies were published on the possible heat tolerance mechanisms (Sharma et al., 2019; Poudel and Poudel, 2020; Sarkar et al., 2021; Ullah et al., 2022). These papers emphasize the role of heat shock proteins (HSP40-100 and smallHSPs), as well as the involvement of enzymatic (superoxide dismutase, ascorbate peroxidase, catalase, glutathione peroxidase, glutathione reductase) and non-enzymatic antioxidants (ascorbic acid, glutathione, tocopherols, carotenoids and phenolic compounds). Other elements of tolerance include the osmolyte adjustment, protein refolding, inhibition of apoptosis and protection of cytoskeleton (Sarkar et al., 2021).

Meiosis is the cornerstone of reproductive processes in eukaryotes (Lenormand et al., 2016). This specialized division of diploid mega- and microspore mother cells yields haploid daughter cells, enabling the start of gametogenesis (Bennett et al., 1973). Since meiosis has a high impact on plant yield it is desirable to reveal the exact mechanisms responsible for its sensitivity to heat stress in cultivated plants besides model species like *Arabidopsis* (Wang et al., 2021). Thus, future tackling of the meiotic process may contribute to crop improvement (Lambing and Heckmann, 2018; Kuo et al., 2021; Fayos et al., 2022).

The plant cytoskeleton is a dynamic filamentous network consisting of microtubules (MTs), actin filaments (AFs), and intermediate filaments (He et al., 2020). This complex system has diverse roles in the life of a cell: it participates in the formation of cell shape, sets up and maintains cell polarity, transports organelles, and coordinates cell division (Kost and Chua, 2002). Correct organization of mitosis and meiosis requires a complex regulatory system that consists of microtubule- and actin filament-associated proteins (Krtková et al., 2016; Paez-Garcia et al., 2018). These proteins are responsible for the controlled polymerization, depolymerization, nucleation, severing, and crosslinking of MTs and AFs. Microtubule organization and spindle dynamics play a crucial role in the correct segregation and movement of chromosomes and the accomplishment of cytokinesis during meiosis (Zamariola et al., 2014; Li et al., 2015). Recent studies revealed the involvement of plant cytoskeleton in abiotic stress response (Kumar et al., 2023). Consequently, the possible detrimental effects on cytoskeletal integrity triggered by high temperature may severely impair the structure and function of daughter cells and, subsequently, the gametes. Depolymerization of microtubules (Smertenko et al., 1997) and actin filaments (Malerba et al., 2010) were observed following *in vitro* heat treatments in tobacco somatic cell suspensions. However, a detailed analysis of heat-triggered effects on the cytoskeleton and the general structure of germline cells is still missing in plants.

The execution of meiosis and the following gametogenesis depends on completing numerous milestones. Chromosome pairing, synapsis- and crossing-over formation occur during the meiotic prophase I, enabling the flawless accomplishment of genetic recombination (Ur and Corbett, 2021). Spindle construction, supervised by the spindle assembly checkpoint (SAC), happens during metaphase. This intricate mechanism ensures the proper segregation of chromosomes, preventing aneuploidy in daughter cells (Motta and Schnittger, 2021).

A plethora of different factors influences the development of pollen grains. Microsporogenesis and microgametogenesis are controlled by several tightly regulated transcription factors (for a review, see Gómez et al., 2015). Another factor for appropriate microspore development is the adequate callose wall formation of tetrads. While exact function of this wall is yet to be revealed, it is generally accepted that normal callose deposition is required for successful microgametogenesis (Dong et al., 2005). Improper building or incorrect dissolution of this callose wall lead to sterility (Zhang et al., 2007; Zhu et al., 2008; Lu et al., 2014). In the male gametophyte, the successful development of gametes from microspore mother cells (MMCs) largely depends on the proper function of the tapetum, which is the innermost cell layer of the anther. This specialized tissue provides nutrients and developmental factors for the microspores and undergoes programmed cell death (PCD) before the completion of gametogenesis (Lei and Liu, 2020). In addition to the nourishing role of tapetum, recent studies indicate that this layer also provides essential regulatory elements, such as small RNAs to complete the male meiotic cycle (Lei and Liu, 2020). Construction of the sporopollenin pollen wall and the pollen coat, important factor of pollen viability is also carried out mainly by the tapetum (Jiang et al., 2013). The undisturbed accomplishment of male meiosis and the following gametogenesis requires a well-regulated network of numerous gene sets, from the meiotic prophase I to the PCD of tapetum cells (Chen et al., 2010; Yamada and Goshima, 2017; Yao et al., 2022). Despite the agronomic significance of wheat, the differential expression of these relevant genes triggered by heat stress has not been described in wheat anthers.

The molecular mechanisms and the signaling of the heat sensitivity observed during gametogenesis are long-standing questions in plant reproduction biology. Despite the crucial role of the cytoskeleton in meiosis, there is no detailed analysis available about heat-driven cytoskeletal changes in meiocytes. Only cytological (Omidi et al., 2014) and histological (Saini et al., 1984; Browne et al., 2021) studies have been made on wheat meiocytes. Transcriptomic investigations on meiosis- and cytoskeleton-specific genes and of genes taking part in tapetal functions are still missing.

Our objectives during the present study were (1) to identify the possible origins of heat-triggered spikelet fertility loss through the structural comparison of meiocytes from heat-sensitive and -tolerant wheat genotypes and (2) to reveal corresponding differential gene expression patterns in these genotypes. Via a combined analysis of morphological and transcriptomic data, our goal was to shed light on the background of meiotic heat sensitivity in the wheat male gametophyte.

## Materials and methods

### Plant material and cultivation

Two winter wheat genotypes were selected for the experiments based on their significantly different spikelet fertility (seed-set) after a meiosis-staged heat-stress treatment, as confirmed in several studies (Végh et al., 2018; Balla et al., 2019; Janda et al., 2019; Marček et al., 2019). The Hungarian ‘Mv 17-09’ genotype was considered sensitive to heat, and ‘Ellvis’, a German cultivar, was taken as heat tolerant. For each experiment, seeds (n=50 per genotype and treatment) were sown in Jiffy peat pellets. Seedlings were subjected to 7 weeks of vernalization at 4°C, then planted in plastic pots containing 2 kg of a soil-sand-peat mixture (3:1:1 parts, by volume). Plants were transferred to growth chambers (Conviron, Winnipeg, Canada) and grown using the T1 spring climatic program (Tischner et al., 1997).

### Developmental staging of meiocytes

Determination of anther developmental stage for treatment and sample collection was based on measurement of flag leaf emergence from the leaf sheath and spike length of main tillers. First, we determined the above mentioned parameters each morning from the beginning of flag leaf emergence on a separate population of plants. Each day, developmental stage of the meiocytes from the measured plants were identified by staining the anthers using 2% acetocarmine according to Shunmugam et al. (2018). Onset of meiosis was considered when meiocytes at the middle region of spikes were at prophase (flag leaf emergence 6 cm, spike length 5 cm). Leaf emergence and spike length characteristic for the day before the onset was determined as well (5 cm and 4.5 cm, respectively) The above mentioned parameters were measured for each plant at each morning, and when the parameters reached the value characteristic for the day before the onset of meiosis, plants were assigned as control or treated (50-50% of the plants). For treated plants, heat stress was immediately started by transferring these plants in a stress chamber. For control plants, cultivation continued as before.

### Stress treatment, sample collection

Heat stress was delivered by exposing the plants to 35 °C for 24 h at a stress chamber. For sample collection, main spikes from treated plants and control plants at the same developmental stage were harvested. Anther collections for cytoskeleton staining and RNA extraction were carried out from the main spikes at the end of the treatments. Anther collections for histology, as well as anther and pistil collections for morphology were carried out from control and treated plants before anthesis when anthers at the middle region of the spike are fully developed and start to turn yellow (on the day before anthesis started). For pollen grain morphology, sample collections were carried out at anthesis (pollen shed). Anthers collected for RNA extraction were immediately placed in RNA*later* stabilization solution (Thermo Fisher Scientific, Waltham, United States) to stabilize and protect RNA. We provide a summary table on sample collection (**Supplementary Table 1**).

### Determination of spikelet fertility rate and yield

Ten plants per genotype and treatment were grown to full maturity, and then the floret number, grain number, and grain yield of the main spikes were determined. The spikelet fertility rate (or seed-set, Prasad et al., 2006) was calculated as follows:


$$\text{Spikelet}\;\text{fertility}\;\text{rate}\;\left(\text{seed}\;\text{set}\right)=\frac{\left(\text{number}\;\text{of}\;\text{filled}\;\text{grains}\right)}{\left(\text{total}\;\text{number}\;\text{of}\;\text{florets}\right)}*100$$

### Histology

For histological studies, anthers were isolated from five plants per genotype and treatment on the day before anthesis, fixed in 50 mM Na-cacodylate buffer (pH 7.2) containing 4% (w/v) formaldehyde at room temperature (RT) for 4 h, washed, dehydrated in an ethanol series, and gradually infiltrated with LR white resin (Ted Pella, Redding, CA, United States). The resin was polymerized at 55°C for 48 h. Semi-thin sections (1 μm) were cut using an Ultracut-E microtome (Reichert-Jung, Heidelberg, Germany) and stained with periodic acid-Schiff (PAS) for polysaccharides and with 1% (w/v) Amido Black for proteins. Stained sections were mounted in DPX (Sigma-Aldrich, 100579) and examined under a DMI-6000 microscope (Leica Microsystems GmbH, Wetzlar, Germany).

### Cytoskeleton labeling and confocal microscopy

To study the cytoskeleton elements, anthers from 20 control and 20 treated plants per genotype were collected at the end of treatment, and the developmental stage was determined by acetocarmine staining of one another per floret. The remaining anthers containing meiocytes at interphase, prophase, metaphase, anaphase and telophase, as well as uninucleate microspores were fixed for 60 min at room temperature (RT) in a microtubule-stabilizing buffer [100 mM 1,4-piperazinediethanesulfonic acid (PIPES), 1 mM MgCl_2_, 2 mM ethylene glycol-bis(β-aminoethyl)-N,N,N′,N′-tetraacetic acid (EGTA), 10% (v/v) dimethyl sulfoxide, 4% formaldehyde, 400 µM 3-maleimidobenzoic acid N-hydroxysuccinimide ester (MBS), 1% (v/v) Triton X-100, and 1.55% (w/v) sucrose, pH 7.0). At the beginning of fixation, anthers were vacuum infiltrated for 2 × 5 min, then washed three times for 10 min in the same buffer with formaldehyde and MBS omitted. Sporogenous archesporial columns containing MMCs were isolated by the MeioCapture method (Shunmugam et al., 2018), and MMCs were settled on adhesive slides for staining. F-actin was stained with Alexa Fluor™ 488-labeled phalloidin (Thermo Fisher Scientific, A12379) at 1:100 dilution for 30 min at RT, mounted in 4’,6-diamidino-2-phenylindole (DAPI)-containing Vectashield Plus medium (Vector Labs, H-2000) and imaged after 15 min.

For microtubule labeling, MMCs were permeabilized in 1% Triton X-100-containing phosphate-buffered saline (PBS), then blocked (1% bovine serum albumin, 0.1 M glycine in PBS) for 30 min. Cells were incubated with a rabbit primary anti-alpha tubulin polyclonal antibody (Agrisera, AS10 680) in 1:500 dilution at RT for 60 min, then at 4°C overnight. MMCs were washed three times in PBS for 30 min, then incubated with an Alexa Fluor 633-labeled anti-rabbit secondary antibody (Thermo Fisher Scientific, A21071) for 60 min at 1:500 dilution. After washing three times, cells were mounted as mentioned before and imaged after 15 min.

The cytoskeleton structure was visualized using a Leica SP8 laser scanning confocal microscope (Leica Microsystems). In the case of F-actin, samples were excited at 488 nm and the emitted fluorescence was detected at 490–550 nm. The microtubule-specific signal was excited at 633 nm, and the signal detected at 640–700 nm. In both cases, DNA was stained with DAPI that was excited at 405 nm and detected at 410-480 nm. *Z*-stack series of MMCs were made at the voxel size of 54 and 200 nm, along the *X*/*Y* and *Z* axes, respectively. Confocal images were deconvoluted using the Huygens v18.04 program and imported in the Imaris v9.8.2 program for 3D reconstruction and cytoskeleton model generation in the case of the following developmental phases: interphase, all phases of meiosis I, and post-meiotic uninucleate microspores (UMs). Three dimensional models of actin filaments and microtubules were generated using the Filaments tool in Imaris (with the following settings: threshold mode, connective baseline=10, minimal ratio of branch length to trunk radius=5. Cumulative lengths of microtubules and actin fibers were automatically measured on the 3D models by the program and were obtained by using the Statistics tab (n=10-16 cells per genotype, treatment, studied phase, and filament type) and expressed as the quantity of cytoskeleton. Since cytoskeletal structures show highly dynamic behavior during the meiotic process (e.g., spindle formation, phragmoplast formation, and expansion), only the MMCs characteristic for each phase were measured to avoid high variation in the data.

### RNA extraction and transcriptome sequencing

For molecular work, all surfaces and equipment were made RNase-free using the RNase AWAY decontamination reagent (Thermo Fisher Scientific, 7002). Anther tissue was ground using a mortar and pestle in liquid nitrogen. Total RNA was extracted using Direct-zol™ RNA MiniPrep Kit, following the manufacturer’s protocol (Zymo Research, Irvine, CA, USA). The RNA concentration and quality were determined by a Qubit 3.0 fluorometer (Thermo Fisher Scientific). Samples having RNA integrity number (RIN) values of at least 7 were used for sequencing. The libraries for RNA-seq were prepared with the NEBNext Ultra II Directional RNA Library Prep Kit for Illumina (NEB, Ipswitch, MA, USA). Briefly, ribosomal RNA was depleted from 400 ng of total RNA using the QIAseq FastSelect rRNA Plant Kit (Qiagen, Hilden, Germany). The depleted RNA was fragmented, end-prepped, adapter-ligated, and the libraries were amplified according to the manufacturer’s instructions. The quality of the libraries was checked on the 4200 TapeStation System using D1000 Screen Tape (Agilent Technologies, Palo Alto, CA, USA), and the quantity was measured on Qubit 3.0. Sequencing was performed on the NovaSeq 6000 system (Illumina, San Diego, CA, USA) with a 2 × 150 bp paired-end run configuration. Every sample had three biological replicates that were sequenced independently.

### RNA-seq analysis

Sequencing results were analyzed by FASTQC v0.11.8 (Babraham Bioinformatics, https://www.bioinformatics.babraham.ac.uk/projects/fastqc/) to assess the read quality. Trimming and adapter removal were performed using CLC Genomics Workbench v23.3 (Qiagen). The trimmed short reads were mapped to the IWGSC RefSeq v2.1 reference genome assembly (Zhu et al., 2021, https://wheat-urgi.versailles.inra.fr/Seq-Repository/Assemblies) by CLC Genomics Workbench v23.3. Expression values were normalized by the TMM method (Robinson and Oshlack, 2010). In order to control the number of potential false positive genes, both Wald test and the likelihood ratio test were applied for the statistical analysis of differential gene expressions, using CLC Genomics Workbench v23.3. *P*-values of both gene lists were adjusted by the FDR *p*-value correction method (Benjamini and Hochberg, 1995). The coding sequences were considered as differentially expressed genes (DEGs) when the FDR *p*-value was lower than 1.0E-6 according to both statistical methods and the normalized fold-change was at least two-fold in absolute value at the same time. Functional annotation of the IWGSC RefSeq v2.1 genome assembly was done by a local BLAST search in the Blast2GO v6.0.3 program (Conesa and Götz, 2008) against the refseq_rna database available at the NCBI website (https://blast.ncbi.nlm.nih.gov/doc/blast-help/downloadblastdata.html#databases) with the following parameter settings: blastn program (-task megablast), E-value cutoff = 1.0E-5. For gene ontology (GO) enrichment analysis, GO terms of DEGs were retrieved by NCBI BLAST search in Blast2GO v6.0.3 using the blastx program against the refseq_protein database at the BLAST expectation value of 1.0E-5, followed by GO mapping and annotation. Overrepresented GO terms of up- and downregulated DEGs were identified in the BiNGO v3.0.5 plugin (Maere et al., 2005) of Cytoscape v3.9.1 program (Shannon et al., 2003), using the hypergeometric statistical test. The whole annotation was used as a reference set, and the *p*-values were adjusted by FDR correction.

### Validation of RNA-seq data was done by quantitative PCR

RNA extraction, DNase treatment, and cDNA synthesis were carried out as described previously (Soós et al., 2012). qPCR was performed on an ABI 7500 Fast real-time PCR System with ABI Fast SYBR Green Master Mix (Thermo Fisher Scientific) according to Soós et al. (2012). Oligonucleotides are listed in **Supplementary Table 2**.

### Identification of wheat ortholog genes

Wheat orthologs of known meiosis-specific genes (Chen et al., 2010) and genes participating in spindle assembly (Yamada and Goshima, 2017) or tapetal functions (Yao et al., 2022) were identified mainly by using the Ensembl Plants database. The conversion between IWGSC RefSeq v1.2 orthologous gene IDs retrieved from Ensembl Plants and the IWGSC RefSeq v2.1 IDs used during mapping and gene expression analysis was done by the ID correspondence table, which can be found in the annotation resources of the IWGSC RefSeq v2.1 genome assembly. The functions of the collected orthologs were verified using our functional annotation carried out by the Blast2GO program. Additional orthologs were identified using the same annotation solely.

### Data handling: statistical analysis and figures

For the assessment of heat stress effects, data were collected for three independent biological replicates. Statistical comparison of spikelet fertility rates, yields, and cytoskeleton measurements of control and treated plants were made by two-way ANOVA and Tukey’s *post-hoc* test (α=0.05) in GraphPad Prism v 10.1.0 program (GraphPad Software Inc., San Diego, CA), with the two main factors tested as genotype and heat stress treatment.

Venn diagrams, horizontal bar plots for enriched GO terms, and bar plots for the fold change expression of the studied gene sets were plotted by SRplot, an online platform for data analysis and visualization (Tang et al., 2023). Box plots were generated using the BoxPlotR web tool (Spitzer et al., 2014).

## Results

### Spikelet fertility showed genotype-dependent reduction after heat stress

**Figure 1:**
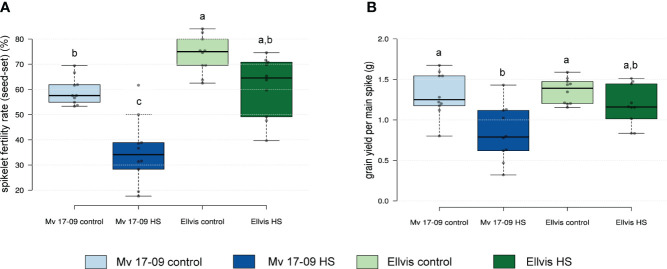
Effect of heat stress on spikelet fertility rate (seed-set) **(A)** and grain yield **(B)** of main spikes in a heat-sensitive (‘Mv 17-09’) and -tolerant wheat (‘Ellvis’) (n=10 per treatment). Different letters above boxes indicate significant differences at least at the P ≤ 0.05 level of probability, according to Tukey’s post-hoc test (α=0.05). Stripes within boxes of the plots indicate median values. Grey dots represent data points.

The spikelet fertility rate in the main spikes of heat-sensitive ‘Mv 17-09’ plants dropped by 40.73%, which was statistically more significant when compared to the 13.91% decrease in the tolerant ‘Ellvis’ (**Figure 1A**). Two-way ANOVA revealed that the effect of genotype and heat treatment were both significant on spikelet fertility, with a significant interaction between these factors (**Table 1**). This indicates that Ellvis showed higher tolerance against heat stress at the time of meiosis. The grain yield of ‘Mv 17-09’ main spikes significantly decreased by 36.96%, while ‘Ellvis’ did not show statistically significant yield loss (**Figure 1B**). According to the results of ANOVA, only the treatment had significant effect on grain yield (**Table 1**).

**Table 1:** Results of the two-way ANOVA analysis carried out on the outcome of spikelet fertility and grain yield per spike studies.

	Two-way ANOVA (P-values)		
Studied parameters	Genotype	Heat Stress	Interaction
Spikelet fertility (seed-set)	<0.0001 ***	<0.0001 ***	0,0418 *
Grain yield per spike	0,1191 ns	0,0004 ***	0,2412 ns

Statistical differences were determined using a two-way ANOVA to examine the effects of genotype, treatment (heat stress) and/or the interaction of these factors. Asterisks denote statistically significant differences (*p<0.05; **p<0.01; ***p<0.001; ns, not significant).

### Anther and microspore development of the sensitive genotype are negatively influenced by heat stress

**Figure 2:**
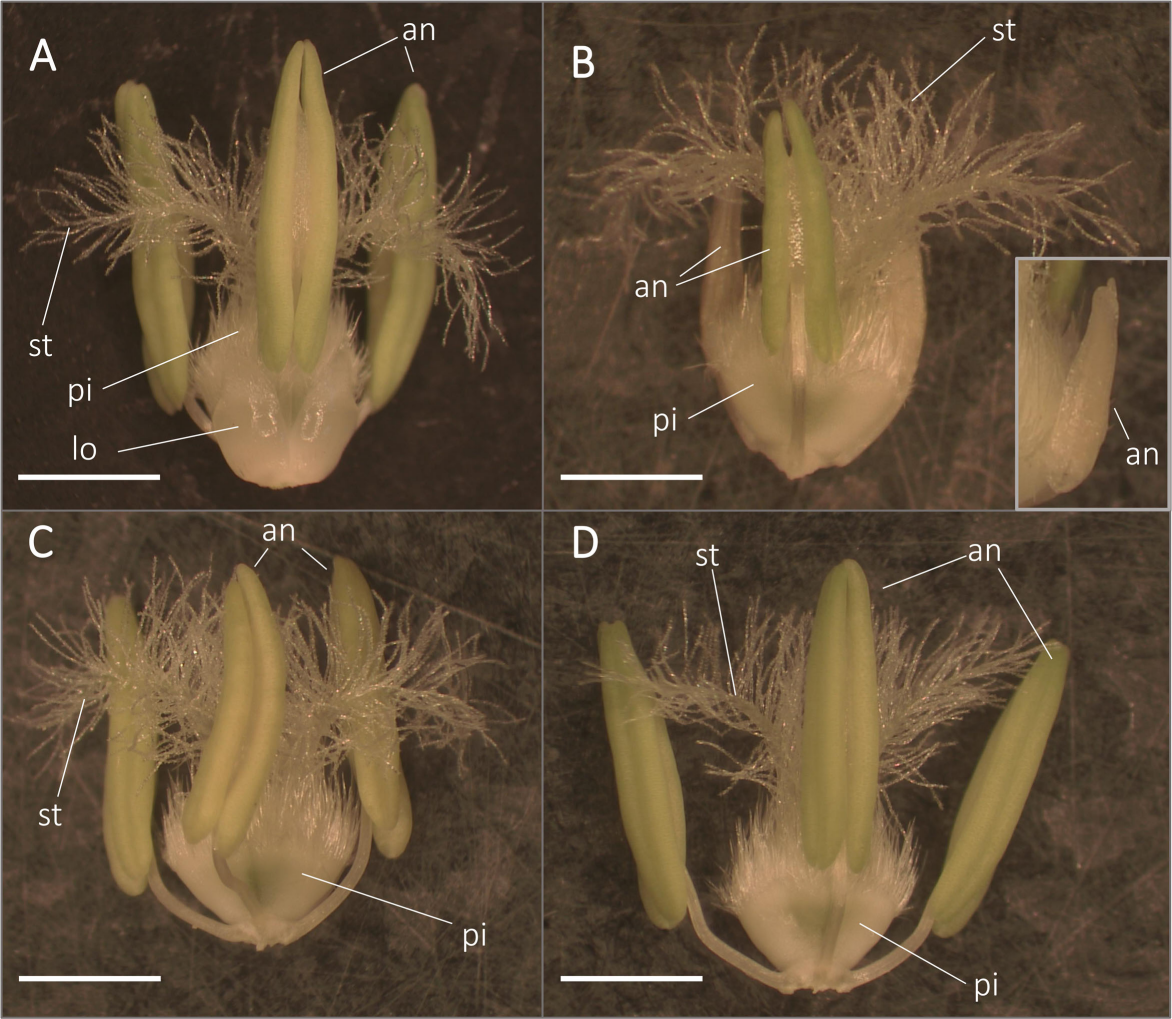
Morphology of anthers and pistils from control **(A, C)** and heat-stressed **(B, D)** ‘Mv 17-09’ **(A, B)** and ‘Ellvis’ **(C, D)** main spikes sampled on the day before anthesis. Note that heat-stressed florets of ‘Mv 17-09’ frequently contained retarded **(B)** and aborted sterile (inset of **B**) anthers. an, anther; lo, lodicule; pi, pistil; st, stigma. Bars represent 2 mm.

**Figure 3:**
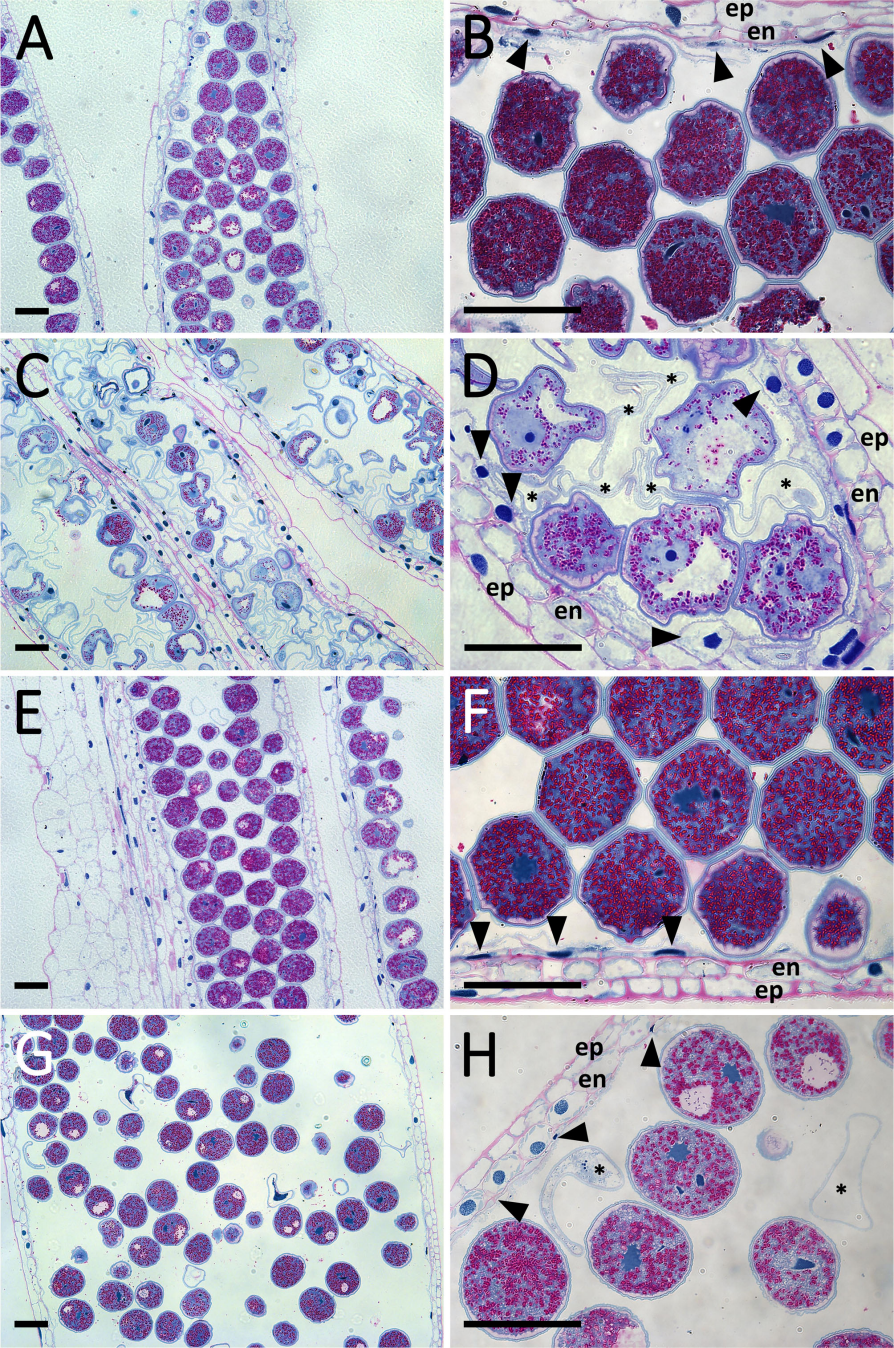
PAS and Amido Black-stained sections of control **(A, B)** and heat-stress treated **(C, D)** ‘Mv 17-09’ as well as control **(E, F)** and heat-stressed **(G, H)** ‘Ellvis’ anthers sampled on the day before anthesis (n= 5 per genotype and treatment). Note the persistent tapetal cells of treated ‘Mv 17-09’ **(D)**. Arrowhead, tapetal cell; asterisk, abortive microspore; en, endothecium; ep, epidermis. Bars represent 50 µm.

Morphological study of florets revealed that pistil and stigma morphology remained normal in both genotypes after the treatment, and heat stress triggered the appearance of retarded and abortive anthers in ‘Mv 17-09’ by the time of anthesis (**Figure 2**). Similar anther wall structure was observed in the control anthers of both varieties, including the degenerated tapetum layer (**Figures 3A, B, E, F**). Although the epidermis and endothecium layers showed normal structure in treated anthers of both genotypes, the development of the tapetum was seriously changed in ‘Mv 17-09’ after heat stress (**Figures 3C, D**). In contrast to the control, tapetal cells remained largely intact, and most of them were cytoplasm-rich, possessing a normally structured nucleus, which suggests that PCD of this layer was delayed significantly (**Figures 3C, D**). A large proportion of microspores was aborted, consisting of only the collapsed wall without cytoplasm (**Figures 3C, D**). Cytoplasm-containing pollen grains exhibited fewer starch grains and larger vacuoles when compared to the control (**Figures 3C, D**; **Supplementary Figure 1**). Treated pollen grains of ‘Ellvis’ did not show serious structural deviations (**Figures 3G, H**). The percentage of structurally abnormal pollen grains (weaker staining, scorched walls, only one nucleus) was determined after pollen shedding, showing a much higher rate of aborted (20.74 ± 5.42 for ‘Mv 17-09’ and 0% for Ellvis) and structurally abnormal (36.62 ± 7.79% for ‘Mv 17-09’ and 6.54 ± 3.62% for Ellvis) pollen grains in the sensitive genotype (**Supplementary Figures 1, 2**).

### Heat stress severely impaired cytoskeleton integrity and chromosome movement during male meiosis

**Figure 4:**
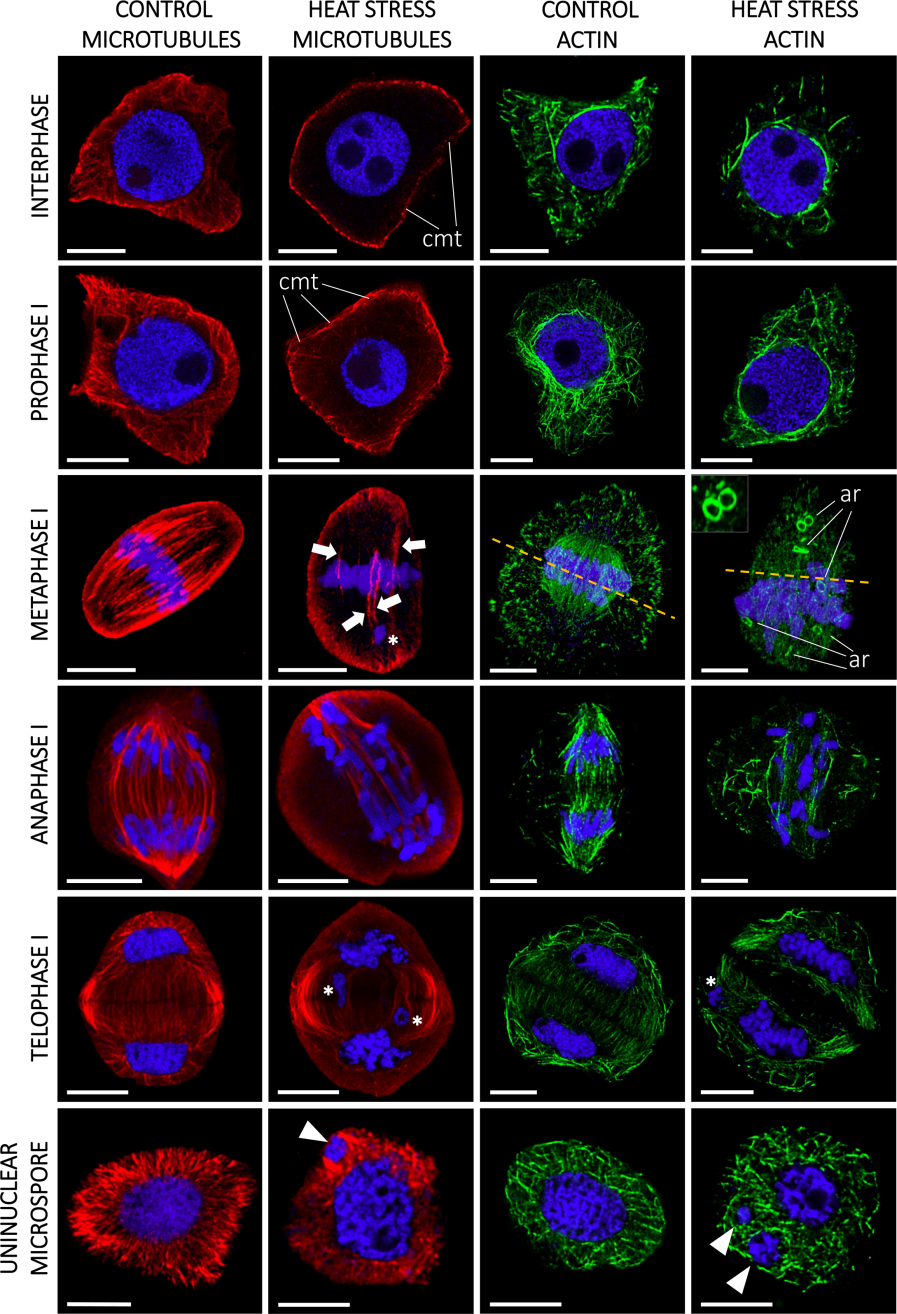
Heat stress significantly altered the microtubule (red) and actin cytoskeleton (green) structure of microspore mother cells (MMCs) during consecutive phases of meiosis and in the uninucleate microspore stage of ‘Mv 17-09’ (n=130). The chromatin is counterstained with DAPI (blue). Note the asynchronous chromosome movement in heat-treated MMCs during anaphase I, which triggered the formation of micronuclei. Arrow, defective spindle filament, arrowhead, micronucleus; asterisk, lagging chromosome; dashed line, symmetry plane; ar, actin ring (see inset, metaphase I); cmt, cortical microtubules. Bars represent 10 µm.

**Figure 5:**
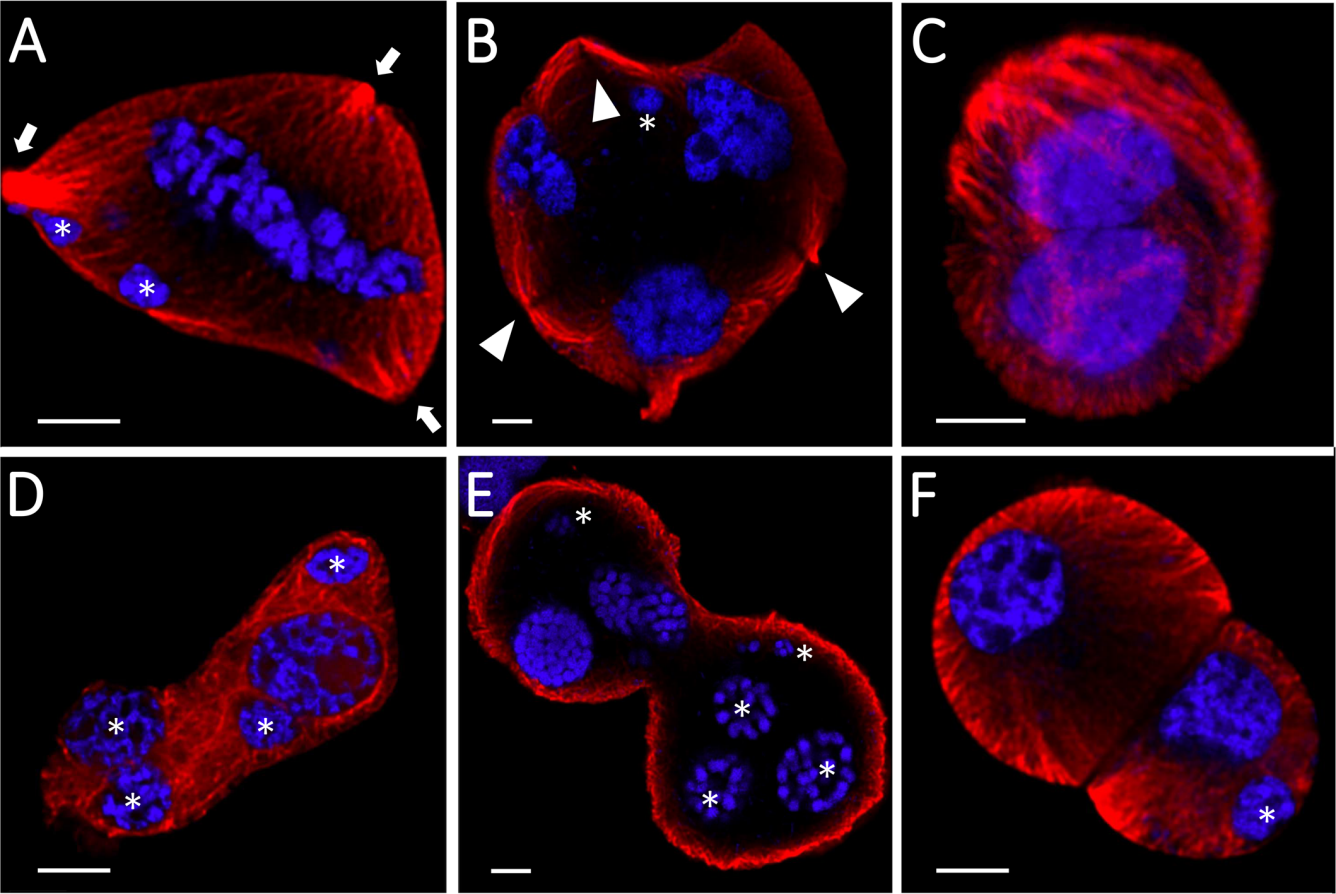
Structural defects of heat-stressed meiocytes and daughter cells of Mv17-09 (n=130). Trilateral symmetry **(A, B)**, nonseparated **(C–E)**, and unevenly divided **(F)** daughter cells. The chromatin is counterstained with DAPI (blue). Arrow, spindle pole; arrowhead, phragmoplast; asterisk, micronucleus. Bars represent 5 µm.

Both genotypes showed altered cytoskeleton morphology, although the impact was more severe in the sensitive ‘Mv 17-09’ during all studied meiotic phases and in daughter cells. In the case of interphase and prophase I MMCs, majority of the microtubules disappeared in the central region of cytoplasm, while, most cortical filaments near the plasma membrane remained intact (**Figure 4**). Depolymerization of actin filaments was observed after heat stress during these phases. Actin bundles located near the nuclei were the most resistant to this effect. Spindle assembly at metaphase I was seriously affected by the treatment, especially in ‘Mv 17-09’. Indirect immunofluorescence assay of the ‘Mv 17-09’ metaphase meiocytes revealed that the formation of kinetochore fibers was severely impaired, causing a narrower, deficient spindle with fewer and/or fragmented fibers (**Figure 4**). Metaphase plate defects occurred frequently, such as improper chromosome disposition outside the plate and asymmetric location of the whole plate. Spindle-associated actin structure was less pronounced, and actin rings were visible throughout the cytoplasm in heat-treated MMCs (**Figure 4**). Chromosome movement was frequently asynchronous during anaphase I of treated meiocytes, leading to multiple lagging chromosomes and later the exclusion of these chromosomes from the forming daughter nuclei at telophase I. The phragmoplast structure showed irregularities during telophase. Micronuclei formed from lagging chromosomes were frequently observed in UMs (**Figure 4**). Perturbed cytoskeleton organization led to various structural defects in meiocytes and daughter cells, such as the formation of tripolar spindle and the resultant trilateral symmetry of the phragmoplast (**Figures 5A, B**), and diverse forms of nonseparated or unevenly divided daughter cells after meiosis I (**Figures 5C–F**). The ratio of structurally damaged meiocytes and UMs counted after heat stress was significantly higher in the case of ‘Mv 17-09’ plants when compared to ‘Ellvis’ (60.76%, n=130 and 32.74%, n=113, respectively, in three independent experiments, *p*=0.039).

### The microtubule cytoskeleton of ‘Ellvis’ showed higher stability after heat stress

**Figure 6:**
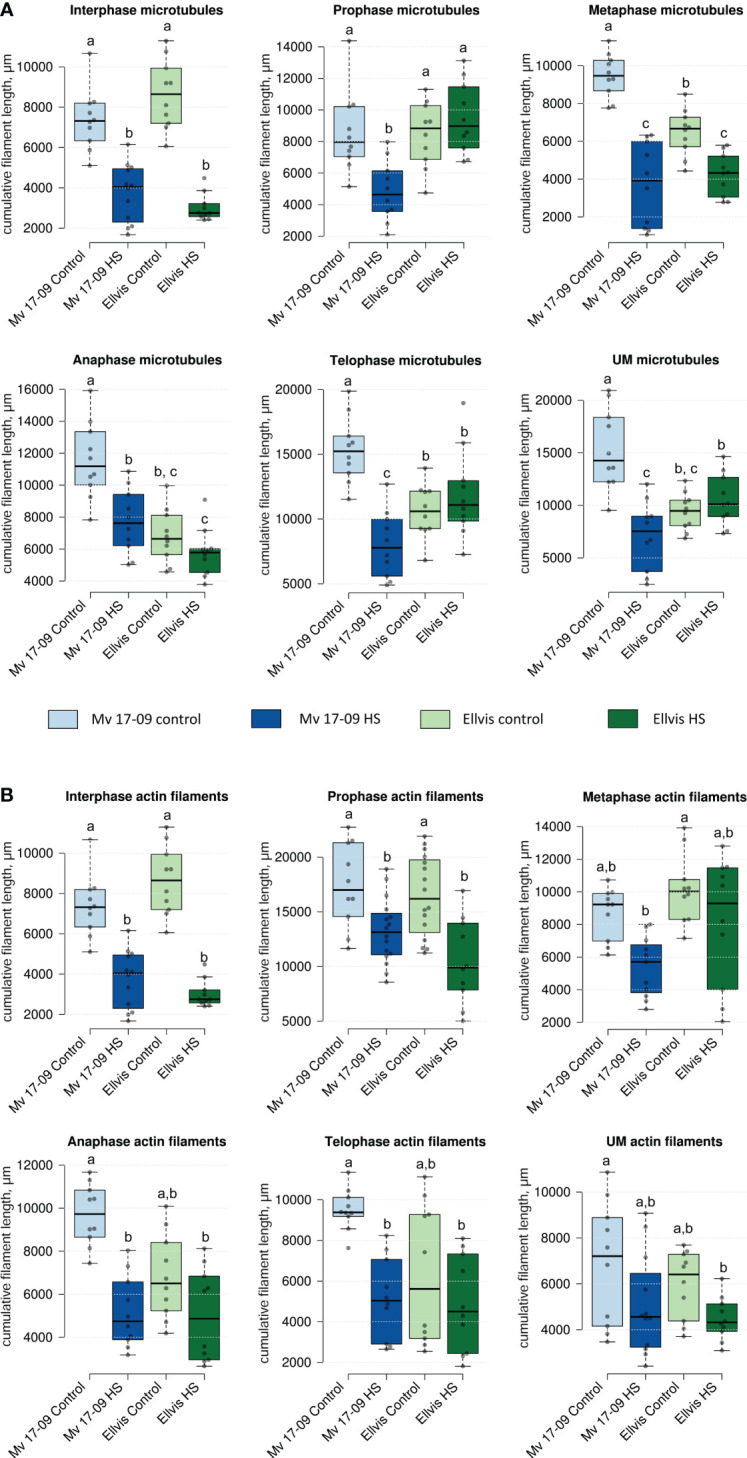
Effect of heat stress on the cumulative length of microtubules **(A)** and actin filaments **(B)** of meiocytes and uninucleate microspores of ‘Mv 17-09’ and ‘Ellvis’ (n=10 to 16 per genotype, treatment and developmental phase). Different letters above boxes indicate significant differences at least at the P ≤ 0.05 level of probability, according to Tukey’s post-hoc test (α=0.05). Stripes within boxes of the plots indicate median values. Grey dots represent data points. ns, not significant; UM, uninucleate microspore.

Quantification of microtubules (**Figure 6A**) and actin cytoskeleton (**Figure 6B**) revealed differential stress responses of ‘Mv 17-09’ and ‘Ellvis’. The cumulative length of microtubules was decreased significantly in all studied phases of meiosis and in the UMs of the sensitive ‘Mv 17-09’ (44.82% at interphase, by 41.70% at prophase, by 58.61% at metaphase, by 31.85% at anaphase, by 48.91% at telophase and by 47.15% at the uninuclear microspore stage). In contrast, this parameter differed significantly in ‘Ellvis’ from the control only at interphase and metaphase I (by 68.15% and 35.11%, respectively; **Figure 6A**). Our results of two-way ANOVA showed that the effect of genotype on microtubule amount was significant only at the prometa- and anaphase, while heat treatment affected the parameter significantly at all studied phases. In addition, the interaction of the studied factors (genotype and heat treatment) significantly influenced the amount of microtubules at all phases, which implies the higher stability of microtubules in Ellvis (**Table 2**). The amount of filamentous actin dropped significantly at interphase and during all phases of meiosis I (by 47.42% at interphase, by 22.68% at prophase, by 38.11% at metaphase, by 51.23% at anaphase and by 46.29% at telophase), while remained constant in UMs in the treated cells of ‘Mv 17-09’ (**Figure 6B**). In the case of ‘Ellvis’, the cumulative length of AFs decreased significantly only at interphase and prophase I (by 60.17 and 38.86%, respectively). Two-way ANOVA revealed that effect of genotype was significant only during interphase and prophase on actin filament amount, while heat treatment influenced this parameter at all phases significantly, with the only exception of metaphase (**Table 2**). Interaction between genotype and treatment factors was significant only at metaphase, which indicates that reaction of actin cytoskeleton to high temperature was basically similar in ‘Mv 17-09’ and Ellvis (**Table 2**).

**Table 2:** Results of the two-way ANOVA analysis carried out on the outcome of microtubule and actin filament cumulative length studies.

	Two-way ANOVA (P-values)		
Studied parameters	Genotype	Heat Stress	Interaction
Interphase MT cumulative length (µm)	0.4514 ns	<0.0001 ***	0.006 **
Prophase MT cumulative length (µm)	0.0003 ***	0.0196 *	0.0001 ***
Metaphase MT cumulative length (µm)	0.0033 **	<0.0001 ***	<0.0001 ***
Anaphase MT cumulative length (µm)	<0.0001 ***	<0.0001 ***	0.0059 **
Telophase MT cumulative length (µm)	0.5198 ns	<0.0001 ***	<0.0001 ***
UM MT cumulative length (µm)	0.0931 ns	<0.0001 ***	<0.0001 ***
Interphase AF cumulative length (µm)	0.0469 *	<0.0001 ***	0.052 ns
Prophase AF cumulative length (µm)	0.0266 *	<0.0001 ***	0.2634 ns
Metaphase AF cumulative length (µm)	0.1862 ns	0.0949 ns	0.0054 **
Anaphase AF cumulative length (µm)	0.5979 ns	0.0005 ***	0.8266 ns
Telophase AF cumulative length (µm)	0.4687 ns	0.017 *	0.7366 ns
UM AF cumulative length (µm)	0.1297 ns	0.0012 **	0.7509 ns

Statistical differences were determined using a two-way ANOVA to examine the effects of genotype, treatment (heat stress) and/or the interaction of these factors. MT, microtubule; AF, actin filament; UM, uninucleate microspore. Asterisks denote statistically significant differences (*p<0.05; **p<0.01; ***p<0.001; ns, not significant).

### Transcriptome analysis and validation

**Figure 7:**
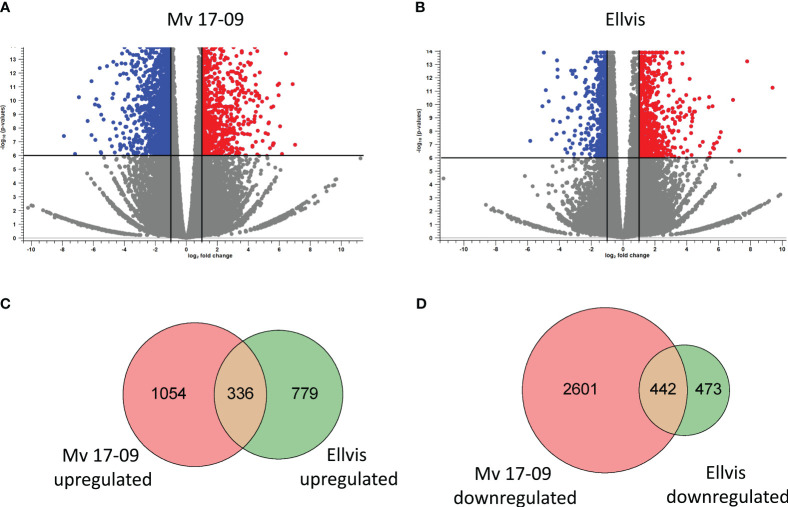
Distribution of significantly (FDR *p*<0.000005, |fold change|≥2) upregulated (red) and downregulated (blue) genes in ‘Mv 17-09’ **(A)** and ‘Ellvis’ **(B)** anthers sampled at the end of treatment. Grey dots represent genes with unchanged expression when compared to the control. Venn diagrams presenting the number of upregulated **(C)** and downregulated **(D)** DEGs in the two wheat genotypes.

**Figure 8:**
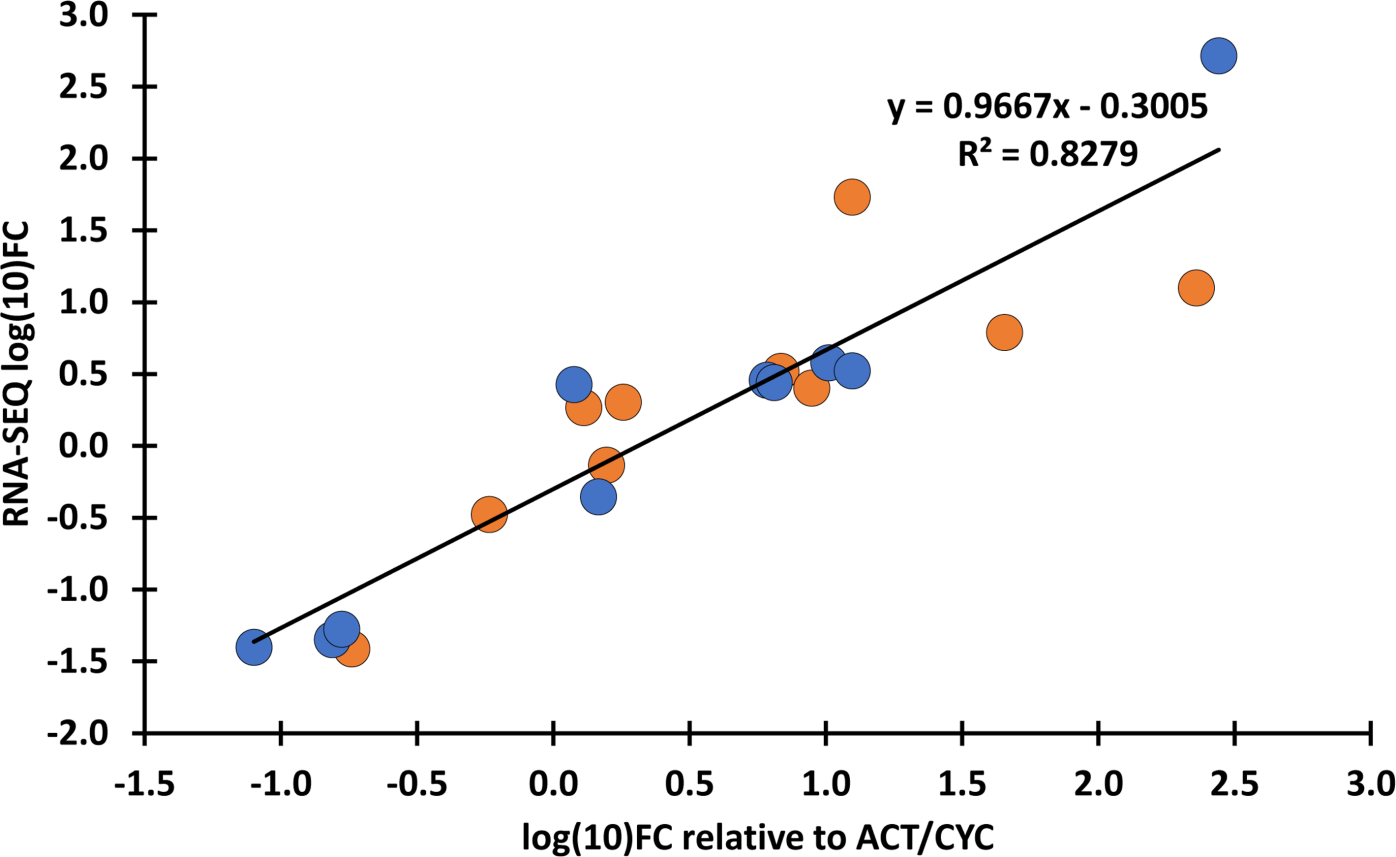
Verification of transcriptome expression data with qPCR. Fold changes (FC) values of the RNA-seq data were compared with the FC calculated as relative expression values of the selected genes to *Ta*ACT3 and *Ta*CYC reference genes as internal controls. Blue: ‘Mv 17-09’; orange: Ellvis. An adjusted coefficient of determination (R^2^) of 0.8279 is observed between the RNA-seq and qPCR data of 10 genes (Pearson’s *r* = 0.9099). qPCR analysis was performed using three independent RNA samples (not the same ones used for RNA-seq). The selected genes, FC values and corresponding primer sequences are listed in **Supplementary Table 2**.

RNA-seq yielded an average of 73 million 150-bp paired-end reads per sample (**Supplementary Table 3**). The GC content of samples ranged from 49% to 52%, and the Phred Q scores in all the samples were higher than 30. Clean reads were aligned to the reference genome assembly at 84.74% to 90.52% efficiency (**Supplementary Table 3**). A total of 4,434 DEGs (1,390 up- and 3,044 downregulated) were identified in ‘Mv 17-09’ and 2,030 DEGs in ‘Ellvis’ (1,115 up- and 915 downregulated), indicating that the rate of downregulated genes was significantly higher after heat stress in the sensitive genotype (**Figure 7**). To validate our RNA-seq dataset, we performed a qPCR study of 10 DEGs on three independent RNA samples (not the same ones used for the RNA-seq). Overall, the fold changes of a panel of DEGs reported by RNA-seq correlated well (*r* = 0.9099) with the fold changes reported by qPCR (**Figure 8**), indicating that the RNA-seq data are robust.

### Gene ontology enrichment analysis

**Figure 9:**
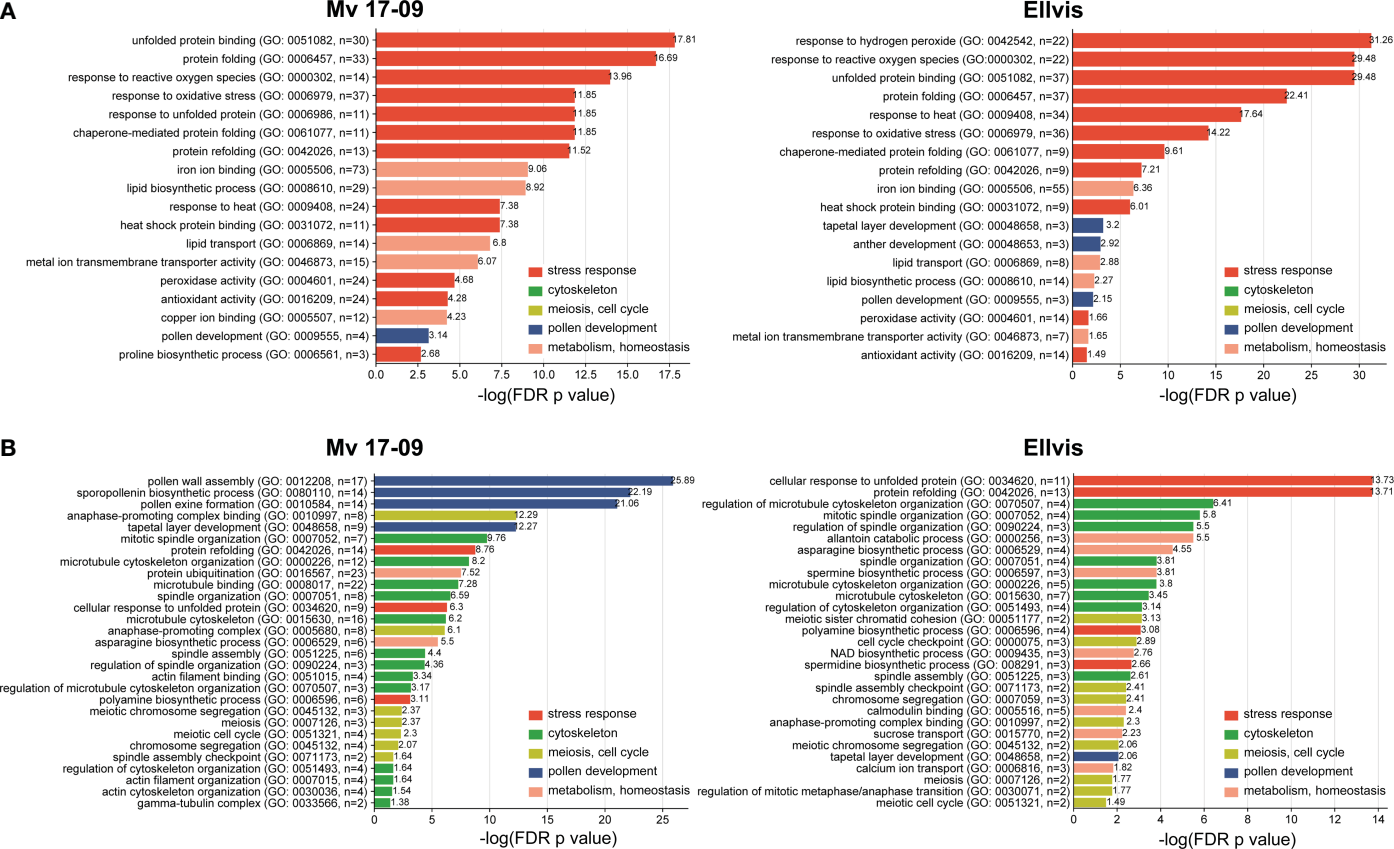
Gene ontology (GO) terms enriched significantly (*p*<0.05) following heat stress among the upregulated **(A)** and downregulated **(B)** DEGs. GO terms are classified based on their functionality.

To shed light on the genome-wide picture of the transcriptomic stress response, gene ontology enrichment analysis was performed on DEGs. We provide a summary of all significantly enriched GO terms at **Supplementary Table 4**. The study revealed that the most highly enriched GO terms among upregulated genes were directly related to stress response in both genotypes, such as response to reactive oxygen species (GO:0000302), unfolded protein binding (GO:0051082), protein folding (GO:0006457), response to oxidative stress (GO:0006979) and response to heat (GO:0009408). All these GO terms showed much higher significance in ‘Ellvis’, confirming the more robust heat-stress response of this cultivar (**Figure 9A**). Other metabolism-related GO terms were enriched in both genotypes, with higher significance in ‘Mv 17-09’, like iron ion binding (GO:0005506); lipid biosynthetic process (GO: 0008610); lipid transport (GO: 0006869) and metal ion transmembrane transporter activity (GO: 0046873) (**Figure 9A**). GO terms associated with pollen development were enriched in greater number in ‘Ellvis’ (3) compared to ‘Mv 17-09’ (1) (**Figure 9A**). Enriched GO terms of downregulated DEGs depicted a radically different stress response between the two genotypes (**Figure 9B**). GO terms associated with the cytoskeleton, meiosis/cell cycle, and microspore/anther development were represented by great numbers and at high significance values in the sensitive ‘Mv 17-09’. In the light of our results regarding MMC and anther structure as well as cytoskeletal organization, the following downregulated terms were the most interesting: spindle organization (GO:0007051), spindle assembly (GO:0051225), anaphase-promoting complex (GO:0005680), pollen grain wall assembly (GO:0010208), tapetal layer development (GO:0048658) sporopollenin biosynthetic process (GO:0080110) and pollen exine formation (GO:0010584). While some of these GO terms were also enriched in ‘Ellvis’, their FDR *p*-values were much lower when compared to ‘Mv 17-09’ (**Figure 9B**). Response to unfolded protein (GO:0006986) and protein refolding (GO:0042026) were enriched among up- and downregulated DEGs in both genotypes, which implies the existence of different gene sets in wheat to cope with protein denaturation under normal and stress conditions. However, ‘Ellvis’ exhibited higher FDR *p*-values regarding these GOs in the case of up- and downregulated DEGs as well (**Figure 9B**).

### Analysis of genes taking part in male meiosis, spindle assembly, and tapetal functions

**Figure 10:**
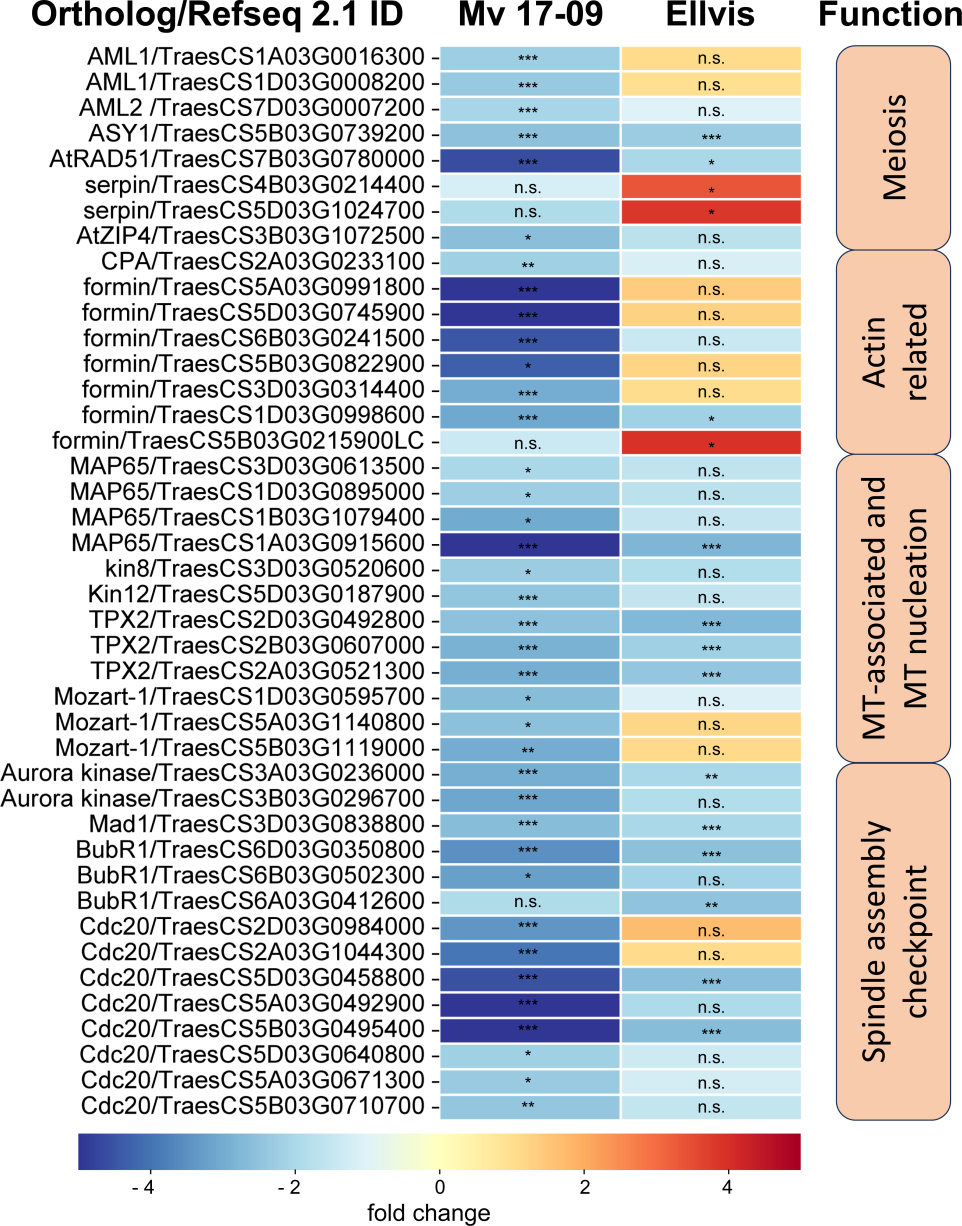
Expression of significant(FDR *p*<1.0E-6, |fold change|≥2) DEGs involved in meiosis, actin nucleation/reorganization and spindle assembly in ‘Mv 17-09’ and ‘Ellvis’anthers sampled at the end of treatment. Asterisks denote statistically significant differences (*p<1.0E-7; **p<1.0E-10; ***p<1.0E-13) between the control and heat stress treated plants of the genotypes. ns, not significant.

The results of the morphological and GO analyses prompted us to study changes in the expression of meiosis-, spindle assembly-, cytoskeleton-, and tapetum-specific gene sets at single-gene resolution. Our search for the wheat orthologs of 67 *Arabidopsis thaliana* genes with known meiosis-related functions (Chen et al., 2010) yielded 209 genes (**Supplementary Table 5**), from which eight were downregulated in ‘Mv 17-09’, while two were up- and also two were downregulated in ‘Ellvis’, respectively (**Figure 10**). The expression of two synapsis-related genes, *Arabidopsis-mei2-like* (*AML*) and *ZIP* decreased only in ‘Mv 17-09’. Two serpin-coding genes showed elevated expression exclusively in ‘Ellvis’ (**Figure 10**).

Yamada and Goshima (2017) provided a list of 92 *Arabidopsis* genes crucial for forming a functional microtubule spindle. We found 240 wheat orthologs of these genes and also added 43 tubulin monomer genes and 26 microtubule severase (*catanin p60* and *p80*, *spastin* and *fidgetin*) genes identified using our BLAST functional annotation (**Supplementary Table 6**). The number of downregulated genes was more than two-fold in ‘Mv 17-09’ (26) when compared to ‘Ellvis’ (only 11), belonging to three categories of functions, namely microtubule-associated proteins, microtubule nucleation and spindle assembly checkpoint proteins (**Figure 10**) No significantly upregulated genes were identified in this gene set. We also studied the expression of 108 actin- and actin-associated proteins (**Supplementary Table 7**) and found that formin genes were the most affected by heat stress, suggesting that actin nucleation was decreased in ‘Mv 17-09’, in contrast to ‘Ellvis’ (**Figure 10**).

**Figure 11:**
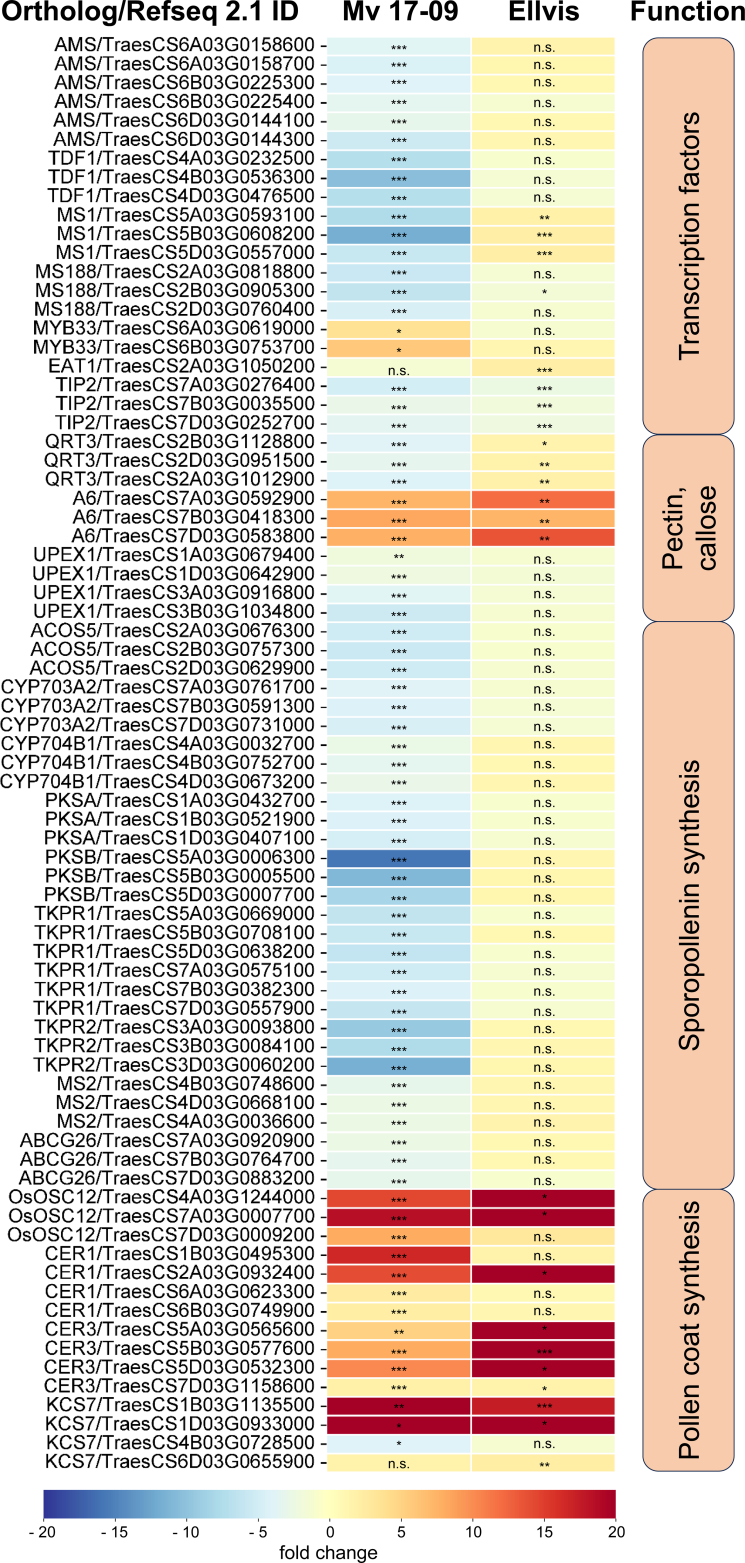
Expression of significant (FDR *p*<1.0E-6, |fold change|≥2) tapetum specific DEGs in ‘Mv 17-09’ and ‘Ellvis’anthers sampled at the end of treatment. Asterisks denote statistically significant differences (*p<1.0E-7; **p<1.0E-10; ***p<1.0E-13) between the control and heat stress treated plants of the genotypes. MT, microtubule. ns, not significant.

The fourth studied gene set was a group of tapetum-specific genes orchestrating the PCD of the tapetum layer and microspore development. We found 202 wheat orthologs of the 36 genes listed by Yao et al. (2022) (**Supplementary Table 8**). The expression of these orthologs was fundamentally different in the genotypes tested here, as these genes were predominantly downregulated in ‘Mv 17-09’ (with 17 up- and 57 downregulated DEGs), while we found only a handful of genes with decreased expression in ‘Ellvis’ (13 up-, and three downregulated orthologs; **Figure 11**). Transcription factors positively regulating the PCD of tapetal cells and genes required for pectin dissolution and sporopollenin synthesis declined in the heat-sensitive genotype while remaining constant in the tolerant ‘Ellvis’. On the contrary, the expression of genes taking part in callose dissolution and pollen coat synthesis was found to be upregulated in both genotypes (**Figure 11**).

## Discussion

### Global transcriptome analysis implicates the key role of cytoskeleton, spindle assembly, and tapetal functions in heat sensitivity

Spikelet fertility and yield data from our experiments confirmed the superior stress-alleviating potential of ‘Ellvis’ over ‘Mv 17-09’. The results of GO enrichment analysis implied various stress reactions behind the differential heat response of the two genotypes. A higher enrichment of upregulated heat- and oxidative stress-related genes, as well as genes related to protein folding and refolding, supported the general stress tolerance of ‘Ellvis’. In addition, we found that the number of downregulated genes was significantly higher after heat stress in the anthers of sensitive ‘Mv 17-09’. This led us to hypothesize that these downregulated gene functions may act behind this differential heat-stress sensitivity. Our GO enrichment analysis revealed that cytoskeleton organization, spindle assembly and tapetum development were the most important downregulated functions which were enriched exclusively, or significantly higher in the sensitive genotype. To draw a detailed picture of the heat-driven impairment during male meiosis and gametogenesis, we investigated the structural deviations, in parallel to expression changes at single-gene resolution.

First, we studied wheat orthologs identified based on meiosis-specific *Arabidopsis* genes (Chen et al., 2010). Although a few synapsis-related genes, such as *Arabidopsis-mei2-like* (*AML*) and *ZIP* (Kaur et al., 2006; De Muyt et al., 2018), were exclusively downregulated in ‘Mv 17-09’, we did not observe structural problems related to this phenomenon. However, serpin genes showed selective upregulation in ‘Ellvis’ anthers. The serpin protease inhibitors are thought to regulate different cell-cycle checkpoints in response to DNA damage (Ahn et al., 2009). Consequently, the upregulation of these genes may improve the stability of the cell cycle at stress. Increased expression of serpins was also observed in heat-stressed durum wheat grains (Laino et al., 2010). *Disruption of meiotic control 1* (*Dmc1*), a meiosis-specific recombinase (Kagawa and Kurumizaka, 2010), was suspected earlier as a meiotic temperature-tolerance gene in wheat, as deletion of its 5D chromosome-specific ortholog was linked to the temperature sensitivity of male meiosis (Draeger et al., 2020). In our experiments, this gene (TraesCS5D02G141200/TraesCS5D03G0353500) did not show significant expression change in either genotype, confirming that meiotic heat sensitivity is a phenomenon with multiple, independent factors involved.

The frequent formation of impaired spindles and the asynchronous movement of chromosomes prompted us to investigate the regulation of cytoskeleton organization and the process of spindle assembly. It was shown previously that the cell division-specific cytoskeletal microtubule structures, such as the spindle and the phragmoplast, exhibit elevated sensitivity to heat stress when compared to cortical microtubules (Smertenko et al., 1997). Our question was whether these structural problems are triggered at least partly by gene expression changes or are induced solely by heat-driven protein denaturation and oxidative stress. Quantitation of MT and AF pools in meiocytes revealed that the cumulative length of both arrays decreased in all phases of meiosis I in ‘Mv 17-09’. However, the mRNA amount of tubulin and actin genes remained the same as in the controls. The dynamics of both networks are orchestrated by a plethora of specific regulatory proteins (Krtková et al., 2016; Paez-Garcia et al., 2018). Decline of cumulative filament length can be caused by depolymerization or decreased nucleation. We found that the expression of neither the MT-severing proteins (severases, like katanin, spastin, and fidgetin, reviewed by Kuo and Howard, 2021) nor the AF-depolymerizing proteins (like actin capping proteins, actin depolymerizing factors (ADFs) or villins (reviewed by de Ruijter and Emons, 1999) showed elevation. In contrast, genes taking part in MT nucleation, such as *mitotic-spindle organizing protein 1* (*Mozart-1*) and several orthologs of the AF-nucleating and -elongation promoting formin genes (Nakamura et al., 2012; Lee and Liu, 2019), were downregulated exclusively in the sensitive genotype. Taken together, silencing of MT and AF nucleation probably contributed to the defective cytoskeletal organization in ‘Mv 17-09’, in contrast to the higher chaperone activity in ‘Ellvis’.

### Downregulation of spindle assembly checkpoint genes may lead to genetically unbalanced pollen grains

Precise execution of male meiosis is ensured through a series of checkpoints. However, these checkpoints are less stringent in plants when compared to other eukaryotes, allowing the generation of genetically unbalanced gametes (Wijnker and Schnittger, 2013). Our structural studies indicated that impairment of the spindle assembly checkpoint (SAC) might be a key event triggering the more frequent formation of micronuclei-containing, functionally damaged microspores in the sensitive genotype. SAC prevents aneuploidy of daughter cells by blocking the onset of anaphase and sister chromatid segregation until all kinetochores are attached properly to the spindle fibers (McAinsh and Kops, 2023). In addition, SAC ensures the correct formation of the metaphase plate, supporting the implementation of equal cell division (Tan et al., 2015). Erroneous operation of SAC leads to asynchronous chromosome movement and lagging chromosomes during anaphase and eventually to the formation of micronuclei. Several genes are known to play a role in this intricate mechanism in model plants (Yamada and Goshima, 2017). Our gene expression study confirmed that numerous orthologs of these genes, such as *Aurora* kinases, *targeting protein for XKLP2* (*TPX2*), *mitotic arrest deficient 1* (*Mad1*), and *Bub1-Related* (*BubR1*), were downregulated in both genotypes after heat stress. *Aurora* kinases are conserved regulators of SAC functions, with three genes in *Arabidopsis*: *Aurora1* and *2* (subgroup A), and *Aurora3* (subgroup B) (Demidov et al., 2005). Though their localizations differ, all of them are suspected to manage the function of other SAC proteins by phosphorylating the H3 histone protein. Reduced expression of these kinases resulted in meiotic defects, aneuploidy, and unreduced pollen grain formation in *Arabidopsis* (Kawabe et al., 2005; Demidov et al., 2014). TPX2 is an important conserved microtubule-associated regulator, acting through the activation of Aurora kinases (Vos et al., 2008; Dvořák Tomaštíková et al., 2020). There are two main protein complexes involved in the SAC mechanism: the anaphase-promoting complex/cyclosome (APC/C) and the mitotic checkpoint complex (MCC) (Hein and Nilsson, 2014). APC/C, a multi-subunit E3 ubiquitin ligase complex, is the effector of SAC, degrading securin and cyclin B, thus promoting the transition to anaphase (Schrock et al., 2020). MCC is another complex of mitotic arrest deficient 2 (Mad2), cell division cycle 20 (Cdc20), BubR1, and *budding uninhibited* by *benzimidazole* (*Bub3*) proteins, which inhibits the activity of the APC/C complex until all chromosomes are properly attached to spindle fibers (Lara-Gonzalez et al., 2021). Although the expression of APC/C subunits did not change after the treatment, several genes of MCC proteins were downregulated. While expression of *BubR1* transcripts decreased in both genotypes, we found that the orthologs of *Cdc20* genes downregulated primarily in the sensitive ‘Mv 17-09’. The role of Cdc20 protein is intriguing in SAC regulation, as it has dual functions in *Arabidopsis* (Lara-Gonzalez et al., 2021). One molecule is needed as an activator of APC/C, taking part in substrate recognition of the complex (Peters, 2006), while another serves as part of the inhibitory MCC complex (Izawa and Pines, 2015). Not surprisingly, a decreased amount of Cdc20 proteins results in abnormal spindle formation, as Niu et al. (2015) found in *Arabidopsis*. According to the Ensembl Plants database, the five existing *Arabidopsis* Cdc20 genes (Cdc 20.1-20.5, Kevei et al., 2011) have 13 orthologues in bread wheat. Expression of these orthologs showed significantly higher stability in the case of ‘Ellvis’, with only two downregulated genes as opposed to eight in ‘Mv 17-09’. Till today, there aren’t any public data on *Cdc20* expression values following heat stress in plant generative cells from any species. However, in *Festuca* leaves, *Cdc20* expression was elevated after 12 h and 36 h of heat stress (Hu et al., 2014), suggesting that the regulation of SAC genes is different between vegetative and generative cells.

### Phragmoplast formation is affected by heat stress

We observed frequent unequal divisions and the failure of cytokinesis in daughter cells. These symptoms implied the perturbance of phragmoplast formation. The phragmoplast is a bipolar array of MTs and AFs responsible for constructing the cell plate in *Arabidopsis* and tobacco (Smertenko et al., 2018) and, subsequently, the new cell wall. We found that two phragmoplast-related proteins, *phragmoplast orienting kinesin 2*, (*POK2*, *kinesin12*) and *microtubule-associated* protein 65-3 (*MAP65-3*), were downregulated because of high temperature, predominantly in the sensitive ‘Mv 17-09’. *POK2* (AT3G19050 in *Arabidopsis*) is a member of the kinesin 12 family and serves as a signal for the correct phragmoplast orientation at the cell cortex (Müller et al., 2006; Lipka et al., 2014). MAP65-3, a 65-kDa MT-associated protein, promotes the recruitment of POK2 to the phragmoplast (Herrmann et al., 2018), thus the appropriate formation of new cell walls. Mutation of *MAP65-3* and the closely related *MAP65-4* genes triggered lethal alterations in gametophytic cell lines of *Arabidopsis* (Li et al., 2017). In a previous study, cold stress was also reported to alter significantly the organization of phragmoplast and cell plate formation in a thermosensitive genic male sterile wheat line (Tang et al., 2011). However, chromosome segregation and tapetal development were unaffected by cold stress.

Although we frequently observed serious structural deviations in the sensitive genotype at the end of heat treatment, pollen grain samples at the time of anthesis contained either dead pollen grains with empty pollen walls or living cells with minor structural alterations. While we did not study occurrence of PCD in developing microspores, it is likely that the microspores containing serious structural defects will undergo PCD and degenerate. It was shown previously that micronuclei degenerate and are eliminated in wheat (Gernand et al., 2005), causing the loss of genetic material. Thus, a fraction of apparently normal mature pollen grains can be functionally defective and contribute to sterility.

### Failure of tapetum degeneration and sporopollenin synthesis contributes to decreased spikelet fertility

It is generally accepted that tapetal dysfunction and/or early tapetal degeneration is a major factor responsible for the low pollen grain viability of heat-treated anthers (Saini et al., 1984; Oshino et al., 2007). As much of the ROS is generated in the mitochondrial respiratory chain, the tapetum, a mitochondria-rich tissue, is highly vulnerable to oxidative stress (Navrot et al., 2007; De Storme and Geelen, 2014). Besides direct damage caused by ROS, such as peroxidation of proteins, nucleic acids, and lipids, these substances may promote premature PCD of the tapetum, triggering the starvation and death of the developing pollen grains in rice (Li et al., 2012; Luo et al., 2013). Other studies, however, point out that failure of tapetum PCD also leads to microspore death and drastically decreased spikelet fertility in *Arabidopsis* and crop plants (reviewed by Lei and Liu, 2020). Li et al. (2011) found that *persistent tapetal cell 1* (*PTC1*), the rice ortholog of the *Arabidopsis male sterility 1* (*MS1*) gene, promotes tapetal PCD and is required for normal microspore development. *MS1* is the final member of a tapetum-specific regulation cascade (consisting of *dysfunctional tapetum* 1 (*DYT1*), *defective in tapetal development and function 1* (*TDF1*), *aborted microspores* (*AMS*), *male sterile 188* (*MS188*), and *MS1* genes), each promoting the expression of the subsequent element. Mutants of these genes showed delayed PCD of tapetum, seriously affecting male spikelet fertility in *Arabidopsis* and rice (Yao et al., 2022). In our study, the expression of these regulators was repressed after heat stress in the sensitive ‘Mv 17-09’, but did not change in ‘Ellvis’. These findings agree with the delayed tapetum degeneration of ‘Mv 17-09’, also revealed by light microscopy (**Figures 3C, D**). The *eternal tapetum1* (*EAT1*) and *TDR interacting protein2* (*TIP2*) transcription factors promote tapetum PCD in rice (Fu et al., 2014; Ko et al., 2014) by upregulating the expression of two aspartic protease genes (Ji et al., 2013; Niu et al., 2013). One *EAT1* ortholog was upregulated in ‘Ellvis’, while *TIP2* orthologs were downregulated in both genotypes. In contrast to our study, it was previously reported that heat stress treatments timed at or right after meiosis resulted in premature tapetal degeneration instead of its persistence in wheat (Saini et al., 1984; Browne et al., 2021). While the applied temperatures were different in these studies (30°C and 35°C, respectively), both groups used a longer, three-day stress treatment. We assume that the longer stress may have triggered the production of higher ROS levels in these experiments, accelerating the degeneration of tapetal cells. In contrast to our results, Browne et al. (2021) did not observe the downregulation of PCD-promoting *AMS* and *myb domain protein8* (*MYB8*) transcription factors in wheat, though they found similar changes to our findings in the case of EAT1 and TIP2 gene expression levels in sensitive genotypes. While our results confirm the previous findings regarding the crucial role of the tapetum in heat-induced sterility, we also clearly showed that failure of tapetal functions can be induced in different ways even by the same kind of stress.

Besides the nourishing function of the tapetum, production of glucanases for callose degradation and tetrade separation is another important task. An important contributor to tetrad separation is the *A6* glucanase gene in *Arabidopsis* (Xu et al., 2016). Wheat orthologs of this gene were found to be upregulated in both genotypes after heat stress. While we did not study the deposition or the correct degradation of callose in tetrad walls by direct methods (such as aniline blue staining), we found that tetrad separation occurred normally after heat treatment. Thus, it can be assumed that elevated expression of *A6* genes did not affect spikelet fertility.

Tapetum-specific sporopollenin synthesis genes were downregulated exclusively in ‘Mv 17-09’, which can be another factor contributing to elevated sterility. It was reported previously that a heat-driven block of sporopollenin synthesis can lead to lethal defects in a temperature-sensitive male sterile wheat line (Yang et al., 2020). In contrast to sporopollenin, pollen coat synthesis genes were upregulated in both genotypes in our experiments. These data suggest that the regulation of tapetal PCD, sporopollenin synthesis, and pollen coat formation are controlled separately, in a genotype-dependent manner.

**Figure 12:**
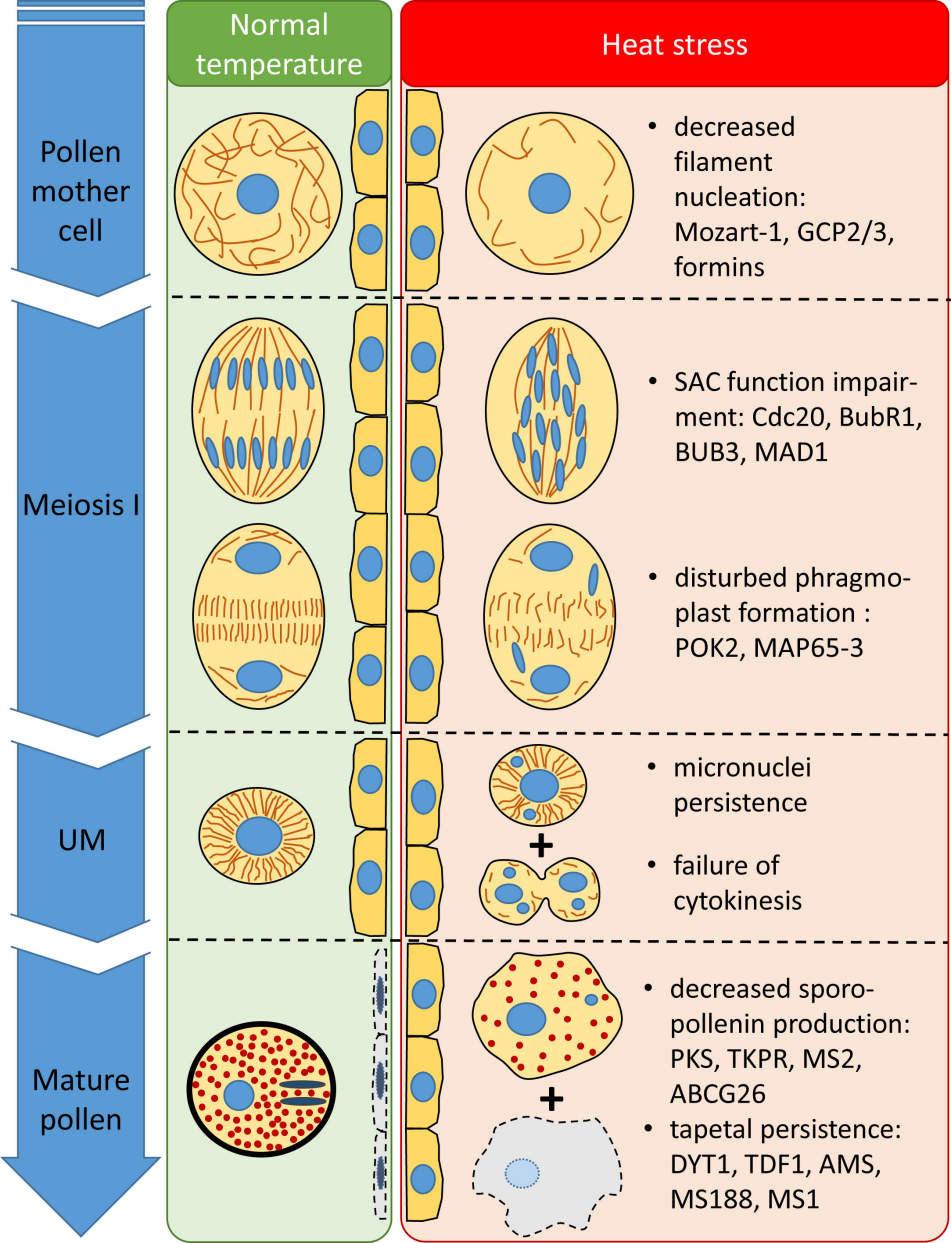
Structural and temporal alterations of wheat male meiocytes and tapetal cells and their supposed transcriptional background triggered by meiosis-timed heat stress.

In our study, heat stress triggered various structural and developmental changes, impairing male fertility. These alterations and their supposed transcriptional background were summarized in **Figure 12**.

## Conclusion

We investigated the factors involved in meiotic heat sensitivity during male gametogenesis of wheat. We demonstrated that even a short exposition to high ambient temperature at the time of meiosis triggers multiple defects in the structure and function of meiocytes. Our results implicate that the failure of the spindle assembly checkpoint and subsequent erroneous chromosome segregation might be the major contributors to spikelet fertility loss via the generation of genetically unbalanced male gametes. In addition, we revealed that heat stress can induce the repression of tapetal degeneration, which is another important factor in pollen grain abortion. Our comparative RNA-seq analysis between heat-sensitive and -tolerant genotypes revealed the transcriptomic basis of these injuries, possibly providing an inventory and baseline of tolerance mechanisms for future breeding efforts. However, further research should be done to reveal the regulatory mechanisms of heat-sensitive gene expression patterns during male gametogenesis.

## Data availability statement

The datasets presented in this study can be found in online repositories. The names of the repository/repositories and accession number(s) can be found below: https://www.ncbi.nlm.nih.gov/geo/, GSE244819.

## Author contributions

AF: Conceptualization, Data curation, Formal analysis, Funding acquisition, Investigation, Methodology, Visualization, Writing – original draft, Writing – review & editing. BP: Investigation, Methodology, Writing – review & editing. VS: Methodology, Validation, Visualization, Writing – review & editing. LS: Formal analysis, Funding acquisition, Visualization, Writing – review & editing, Writing – original draft.
