# Supplementary material for: Heat stress during male meiosis impairs cytoskeletal organization, spindle assembly and tapetum degeneration in wheat
Source: Front Plant Sci. 2024 Jan 8;14:1314021. doi: 10.3389/fpls.2023.1314021 (PMC10800805; doi:10.3389/fpls.2023.1314021)
Supplement: Supplementary file 1 [file DataSheet_1.pdf]

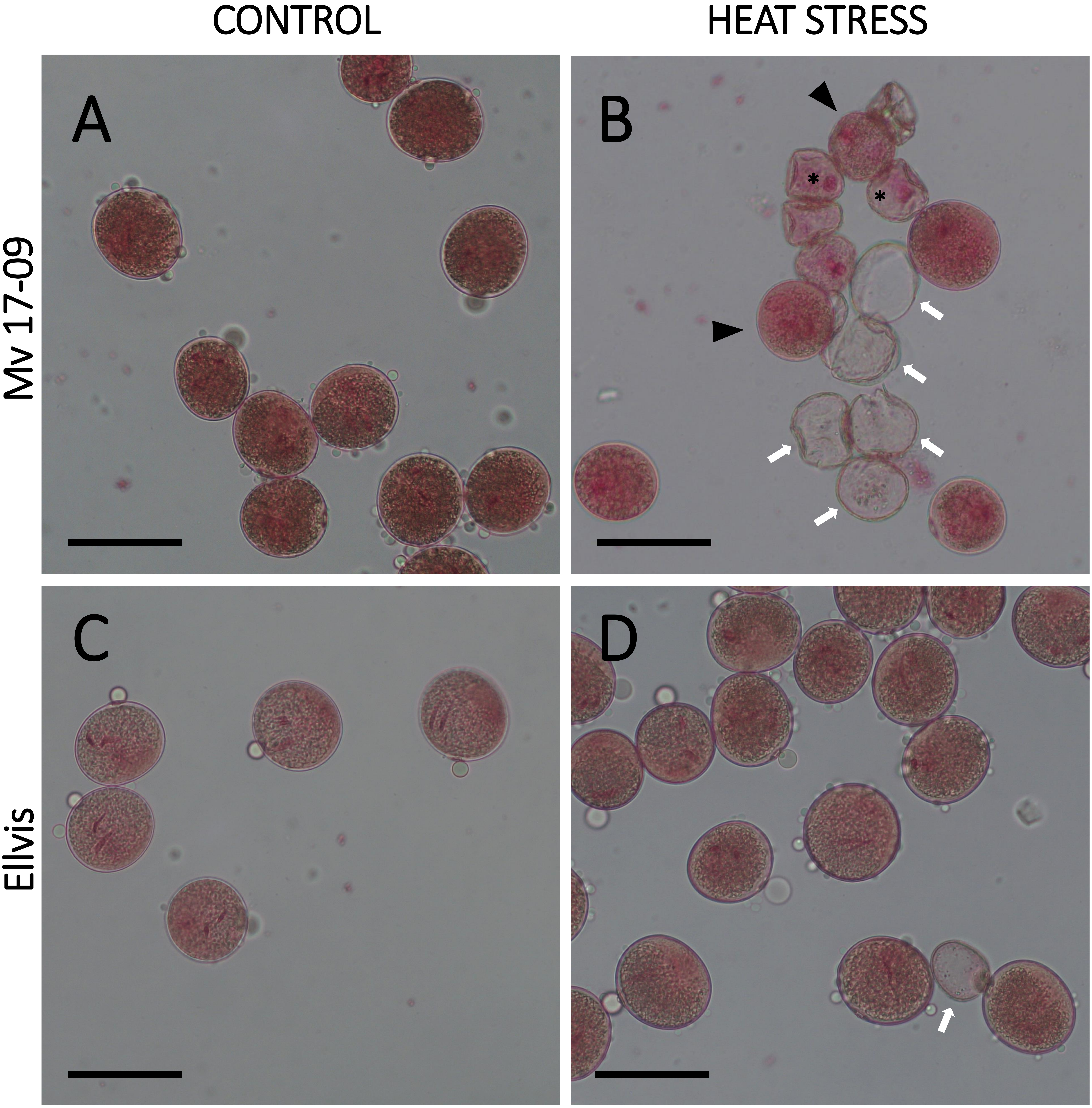

Supplementary figure 1. Morphology of pollen grains after pollen shed from control **(A, C)** and heat-stressed **(B, D)** Mv 17-09 **(A, B)** and Ellvis **(C, D)** main spikes. Arrowhead, retarded pollen grains with low starch content; asterisk, cells containing a single nucleus; arrow, abortive pollen grains. Bars represent 50 µm.
