# Supplementary material for: Heat stress during male meiosis impairs cytoskeletal organization, spindle assembly and tapetum degeneration in wheat
Source: Front Plant Sci. 2024 Jan 8;14:1314021. doi: 10.3389/fpls.2023.1314021 (PMC10800805; doi:10.3389/fpls.2023.1314021)
Supplement: Supplementary file 2 [file DataSheet_2.pdf]

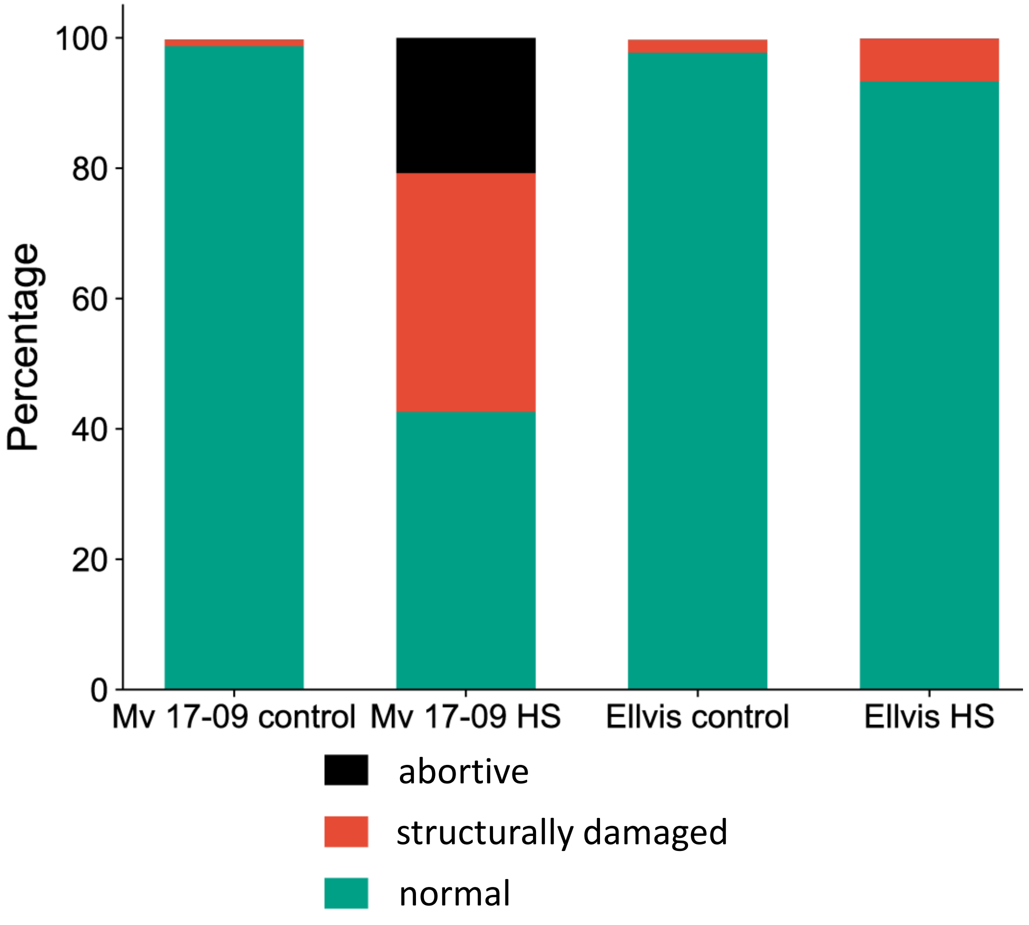

Supplementary Figure 2 Frequency of normal and abnormal pollen grains (n=1200-1800 per genotype and treatment) sampled after pollen shed. HS, heat stress.
